# Validation of algorithms to identify elective percutaneous coronary interventions in administrative databases

**DOI:** 10.1371/journal.pone.0231100

**Published:** 2020-04-07

**Authors:** Catherine G. Derington, Lauren J. Heath, David P. Kao, Thomas Delate

**Affiliations:** 1 Pharmacy Department, Kaiser Permanente Colorado, Aurora, CO, United States of America; 2 Department of Clinical Pharmacy, University of Colorado Skaggs School of Pharmacy and Pharmaceutical Sciences, Aurora, CO, United States of America; 3 Department of Pharmacotherapy, University of Utah College of Pharmacy, Salt Lake City, UT, United States of America; 4 Cardiac and Vascular Center, University of Colorado Health, Aurora, CO, United States of America; 5 Department of Cardiology, University of Colorado School of Medicine, Aurora, CO, United States of America; Erasmus Medical Center, NETHERLANDS

## Abstract

**Background:**

Elective percutaneous coronary interventions (PCI) are difficult to discriminate from non-elective PCI in administrative data due to non-specific encounter codes, limiting the ability to track outcomes, ensure appropriate medical management, and/or perform research on patients who undergo elective PCI. The objective of this study was to assess the abilities of several algorithms to identify elective PCI procedures using administrative data containing diagnostic, utilization, and/or procedural codes.

**Methods and results:**

For this retrospective study, administrative databases in an integrated healthcare delivery system were queried between 1/1/2015 and 6/31/2016 to identify patients who had an encounter for a PCI. Using clinical criteria, each encounter was classified via chart review as a valid PCI, then as elective or non-elective. Cases were tested against nine pre-determined algorithms. Performance statistics (sensitivity, specificity, positive predictive value, and negative predictive value) and associated 95% confidence intervals (CI) were calculated. Of 521 PCI encounters reviewed, 497 were valid PCI, 93 of which were elective. An algorithm that excluded emergency room visit events had the highest sensitivity (97.9%, 95%CI 92.5%-99.7%) while an algorithm that included events occurring within 90 days of a cardiologist visit and coronary angiogram or stress test had the highest positive predictive value (62.2%, 95%CI 50.8%-72.7%).

**Conclusions:**

Without an encounter code specific for elective PCI, an algorithm excluding procedures associated with an emergency room visit had the highest sensitivity to identify elective PCI. This offers a reasonable approach to identify elective PCI from administrative data.

## Introduction

In 2014, approximately 480,000 percutaneous coronary interventions (PCI) and over 1 million inpatient diagnostic cardiac catheterizations were performed in the United States [1]. PCI remains a pillar of treatment for patients who require revascularization in the setting of acute coronary syndromes [2–4]. Additionally, PCI may be used electively to reduce anginal symptomatology or risk for a cardiovascular event in patients with high-risk lesions who are optimized on medical therapy [5]. Studies on elective PCI have largely been conducted within prospective registries or clinical trials, but outcomes of PCI derived from real-world data (i.e., electronic health record or administrative data) are lacking. This is possibly due to a lack of diagnostic and procedural codes that distinguish between elective and non-elective PCI.

Elective PCI remains a controversial treatment for patients diagnosed with chronic stable angina. Results of both the COURAGE and ORBITA trials demonstrated no benefit for mortality, myocardial infarction, or exercise time in patients randomized to elective PCI compared to medical therapy alone and a sham procedure, respectively [6,7]. In addition, elective PCI is an attractive target for national payers to simultaneously reduce cost, optimize quality, and augment patient satisfaction by implementing a “same-day discharge” strategy [8–10]. With the emphasis on outcomes, quality of care, patient satisfaction, and costs, an elective PCI cohort developed in a database that reflects real-world use is necessary to enable comparative effectiveness analyses of management strategies for this high-risk patient population. The ability to identify and track patients who undergo elective PCI in real-world data would enable individual institutions to track and research health outcomes associated with elective PCI, particularly those institutions that do not contribute to national registries like the American College of Cardiology’s National Cardiovascular Data Registry (NCDR). Additionally, the NCDR only links to Medicare fee-for-service data; thus, the ability is limited to evaluate outcomes on non-Medicare populations with this registry.

To our knowledge, no literature exists regarding the development of a validated elective PCI cohort using administrative data. The objective of this study was to develop, validate, and contrast algorithms that use diagnostic, utilization, and/or procedural codes to discriminate elective PCI procedures from non-elective PCI procedures using administrative data.

## Methods

### Study design and setting

This was a retrospective study of adult patients ≥ 18 years of age who underwent a PCI procedure between 1/1/2015 and 6/30/2015 or 1/1/2016 and 6/30/2016. The study was conducted at Kaiser Permanente Colorado (KPCO), an integrated healthcare delivery system providing care to over 650,000 members in urban and rural areas of Colorado in the United States. A random sample of patients who had an encounter with a Current Procedural Terminology (CPT) code for a PCI performed in an ambulatory surgery center (ASC), emergency department (ED), or hospital during the study periods was obtained from an administrative encounter database. Procedures were then assessed via manual chart review by trained abstractors for 1) confirmation that a PCI was actually performed, and 2) whether the PCI was elective or non-elective. Algorithms based on diagnostic, utilization, and/or procedural codes were then applied to assess their abilities to discriminate between elective and non-elective PCI.

KPCO operates 29 medical offices with embedded pharmacies and utilizes an electronic health record (EHR) where information on diagnoses and surgical procedures are documented. In addition, KPCO operates two ASC where patients receive same-day surgical care, including diagnostic and preventive procedures. Furthermore, KPCO contracts with local facilities to provide ED and inpatient services. Coded and free-text medical, pharmacy, laboratory, ED, hospitalization, and membership information from within the delivery system, as well as from contracted and non-contracted facilities, are captured in KPCO’s electronic administrative and claims databases. At the time of the study, there were no KPCO guidelines to direct the appropriateness of performing an elective PCI. All aspects of this study, including non-anonymized medical record review, were reviewed and approved by the KPCO Institutional Review Board with a waiver of informed consent. Because the data used for this study contain business-sensitive information, the data and study materials will not be made available to other researchers.

### Patient population

All adult patients who had an encounter with at least one CPT code (92920–92924, 92928, 92929, 92933, 92934, 92937, 92938, 92941, 92943, and 92944) for a PCI procedure between 1/1/2015 and 6/30/2015 or 1/1/2016 and 6/30/2016 were identified from an administrative database. Time frames around the October 2015 transition from the *International Classification of Diseases*, *Ninth Edition* (ICD-9) to the *International Classification of Diseases*, *Tenth Edition* (ICD-10) were used to increase the generalizability to broad clinical and research settings [11,12]. The index date was the date of the included PCI procedure. Patients undergoing multiple PCI within the study time periods were included if the PCI were at least one day apart. Therefore, a patient could be included multiple times in the study. PCI were chart reviewed to confirm as true PCI. If unable to confirm as a true PCI or classify as elective or non-elective, the procedure was excluded from analysis.

### Classification of PCI procedures

A sample of 500 PCI procedures were randomly selected for chart review. There is no accepted standard for the number of events to review for a validation study such as this; therefore, 500 was chosen for reviewing as this count of medical charts was feasible to review with the available resources at the time of the study. The randomization procedure was not enriched for codes that may increase or decrease the number of elective PCI cases (i.e., all codes were given equal weight). Two clinicians (C.G.D. and L.J.H.) were trained to abstract information from the EHR and categorize each procedure as a true PCI and then as “elective” or “non-elective.” Definitions were determined *a priori* with input from the study cardiologist (D.P.K.) and adjusted throughout the course of chart reviews, as needed, with discussion and consensus between the investigators.

Hospitalization, ED, procedure, clinical, and telephone practitioner notes documented in the EHR were used to validate each procedure. Procedures were considered elective if the terms “elective,” “non-primary,” “non-emergent,” or “staged” were used in any of the documentation. Procedures were considered non-elective if the terms “emergent,” “primary,” “urgent,” “immediate,” “ASA Status 5E,” “cardiac alert,” or “cardiac arrest” were used. If none of these terms were used, a set of clinical criteria informed by current guidelines [2–4,13], appropriate use criteria [5], and input from the study cardiologist, was used to determine if a procedure was non-elective. These clinical criteria included: ruptured plaque, stent thrombosis, hemodynamic instability (i.e., tachycardia, bradycardia, hypotension, shock), any myocardial infarction, unstable angina, troponin values greater than 0.5 ng/mL, or chest pain (at rest or with accelerating tempo and/or severity). In the case that no consensus could be reached, a third abstractor (T.D.) acted as a tiebreaker.

To assess the inter-rater reliability, 20 identical PCI initially were reviewed independently by each abstractor. The kappa statistic indicated strong agreement (K = 0.85, 95% CI 0.59, 1.00). For the study, each reviewer assessed 250 procedures; however, due to limited information in the EHR for 21 procedures, an additional 21 procedures were selected for review.

### Algorithms

**Fig 1:**
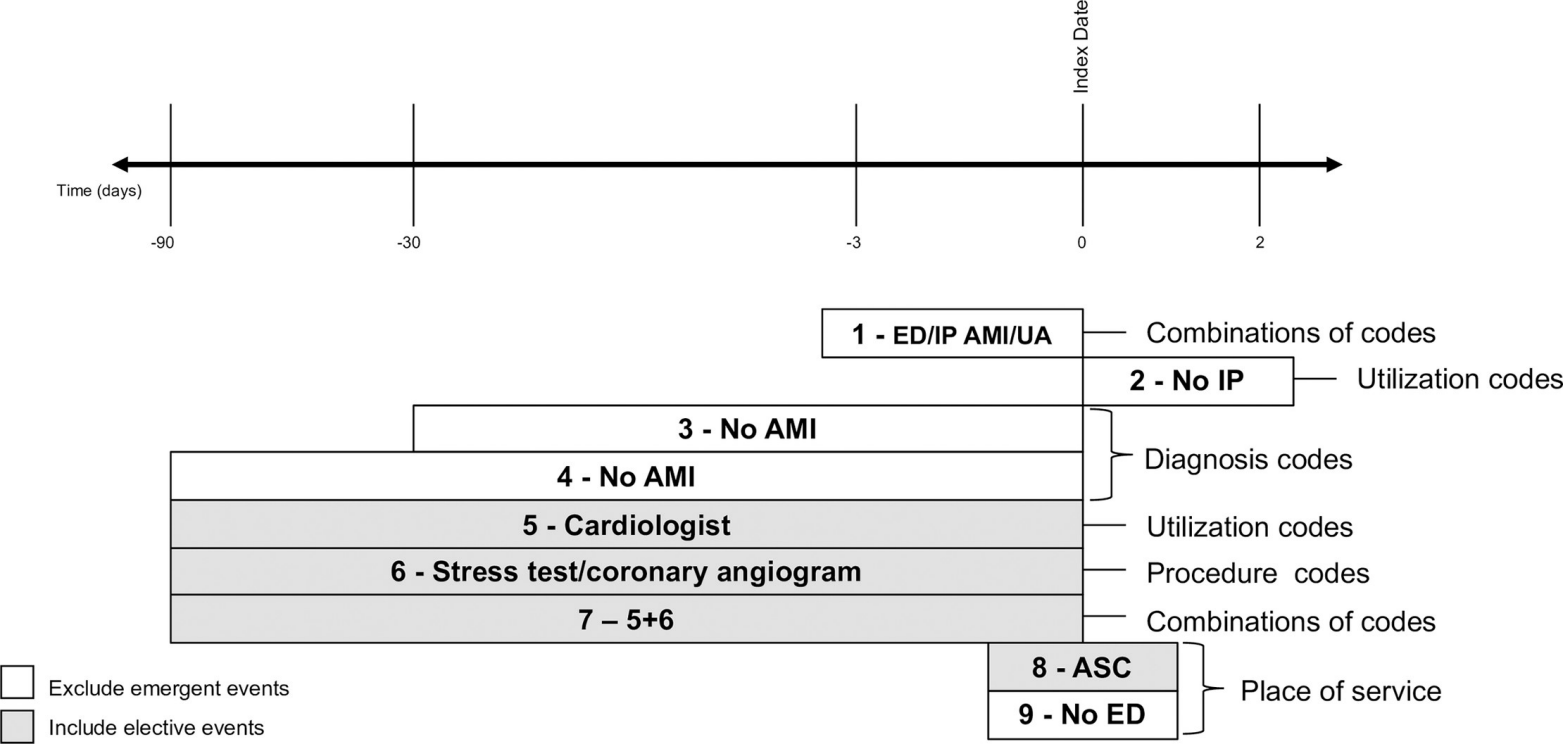
Description of algorithms relative to the index PCI event. Each algorithm was defined by diagnosis codes, utilization codes, procedure codes, or place of service codes alone or in combination relative to the date of the PCI (Day Zero on timeline; see Methods). The algorithms were designed to either exclude emergent events (white boxes) or include elective events (grey boxes). ASC = ambulatory surgery center; AMI = acute myocardial infarction; ED = emergency department; IP = inpatient; PCI = percutaneous coronary intervention; UA = unstable angina.

After each procedure was categorized as elective or non-elective, nine algorithms determined *a priori* were tested against the sample. These algorithms were developed with the purpose of identifying an elective PCI using administrative data. They varied in terms of time from index date with combinations of diagnosis codes, healthcare utilizations (i.e., office visits and hospitalizations), procedure setting (i.e., ambulatory or inpatient), and specific procedures (Table 1 and Fig 1). The algorithms are broadly defined into 4 groups. Algorithms 1 and 2 assessed for ED or hospitalizations for acute myocardial infarction or unstable angina relative to the index date using ICD-9 (410.x, 411.1, 413.0, 411.81, 411.89) or ICD-10 diagnosis codes (I21.x-I23.x, I20.0, and I24.x). Algorithms 3 and 4 assessed for documentation of an acute myocardial infarction ICD-9 or ICD-10 coded with any encounter within the 30 and 90 days prior to the index date (same codes as above). Algorithms 5 through 7 assessed for either an ambulatory cardiology visit, diagnostic procedure (i.e., stress test or coronary angiography), or combination of the two within the 90 days prior to the index date. CPT codes were used for diagnostic procedures (92978, 92979, 93454, 93463, 93464, 93571, and 93572). Algorithms 8 and 9 assessed the acuity of the setting where the associated PCI procedure occurred; algorithm 8 assessed whether the encounter place of service was an “ambulatory” visit (as would occur for routine, low-risk outpatient procedures such as an elective PCI) while algorithm 9 defined procedures where the place of service was not an emergency visit (e.g., nursing homes, skilled nursing facilities, hospice, telephone encounters) (as would occur for emergent PCI cases). The places of service are mutually exclusive of one another in the data but not inclusive of other settings; therefore, separate algorithms were needed.

**Table 1:** Algorithm definitions.

Algorithm No.	Definition	Types of Codes Used
1	No emergency department visit or inpatient stay for acute myocardial infarction (AMI) or unstable angina in the three days prior to PCI	Combinations of codes
2	No subsequent hospitalization for longer than two days immediately after PCI	Utilization codes
3	No AMI ICD-9/10 code in prior 30 days	Diagnosis codes
4	No AMI ICD-9/10 code in prior 90 days	Diagnosis codes
5	At least one outpatient cardiology provider visit in prior 90 days	Utilization codes
6	Stress test and/or angiography CPT code in prior 90 days	Procedure codes
7	At least one outpatient cardiology provider visit and either stress test or angiography CPT code in prior 90 days	Combinations of codes
8	Coded as an ambulatory visit	Place of service
9	Not coded as an emergency department visit	Place of service

AMI = acute myocardial infarction; CPT = current procedural terminology; ICD = *International Classification of Diseases*; PCI = percutaneous coronary intervention

### Data analysis

Age was calculated as of the index date. The Chronic Disease Score, a measure of a patient’s chronic illness burden, was calculated from ambulatory medication dispensings recorded during the 180 days prior to the index date [14]. A Charlson Comorbidity Index was calculated from diagnoses that were recorded during the 180 days prior to the index date [15]. Patient characteristics were reported as means ± standard deviations (SD) for normally-distributed data and medians [interquartile ranges, IQR] for non-normally-distributed data, with appropriate tests used to assess for significant differences between the groups (e.g., chi-square test of association, t-test, Wilcoxan rank-sum test).

The results of each algorithm (yes/no the criteria were met) were compared to the chart-review validation gold standard of elective PCI to calculate the sensitivity, specificity, positive predictive value (PPV), and negative predictive value (NPV) for each algorithm. No specific threshold for any of these performance metrics formally exists to define the adequacy of an algorithm’s performance, as these measures are sensitive to the population in which the metrics are evaluated [16]. Previous validation studies have evaluated algorithms with >80% performance as “adequate” for a variety of codes and clinical conditions (e.g., stroke, heart failure, rheumatoid arthritis) [17–20]. We also performed a sensitivity analysis for the algorithms that used diagnosis codes (algorithms 1, 3, and 4), stratifying the analysis by date (before and after October 1, 2015) to assess accuracy of the algorithms based on ICD-9 versus ICD-10 codes.

The 95% confidence interval (CI) for each performance statistic was calculated using the Wilson Score Method [21,22]. Given that PPV is highly influenced by prevalence and there is a need to identify pragmatically true elective PCI cases, sensitivity was the primary outcome. An alpha level of 0.05 was used for significance. All analyses were conducted using SAS version 9.4 (SAS Institute, Cary, NC, USA) [23].

## Results

**Fig 2:**
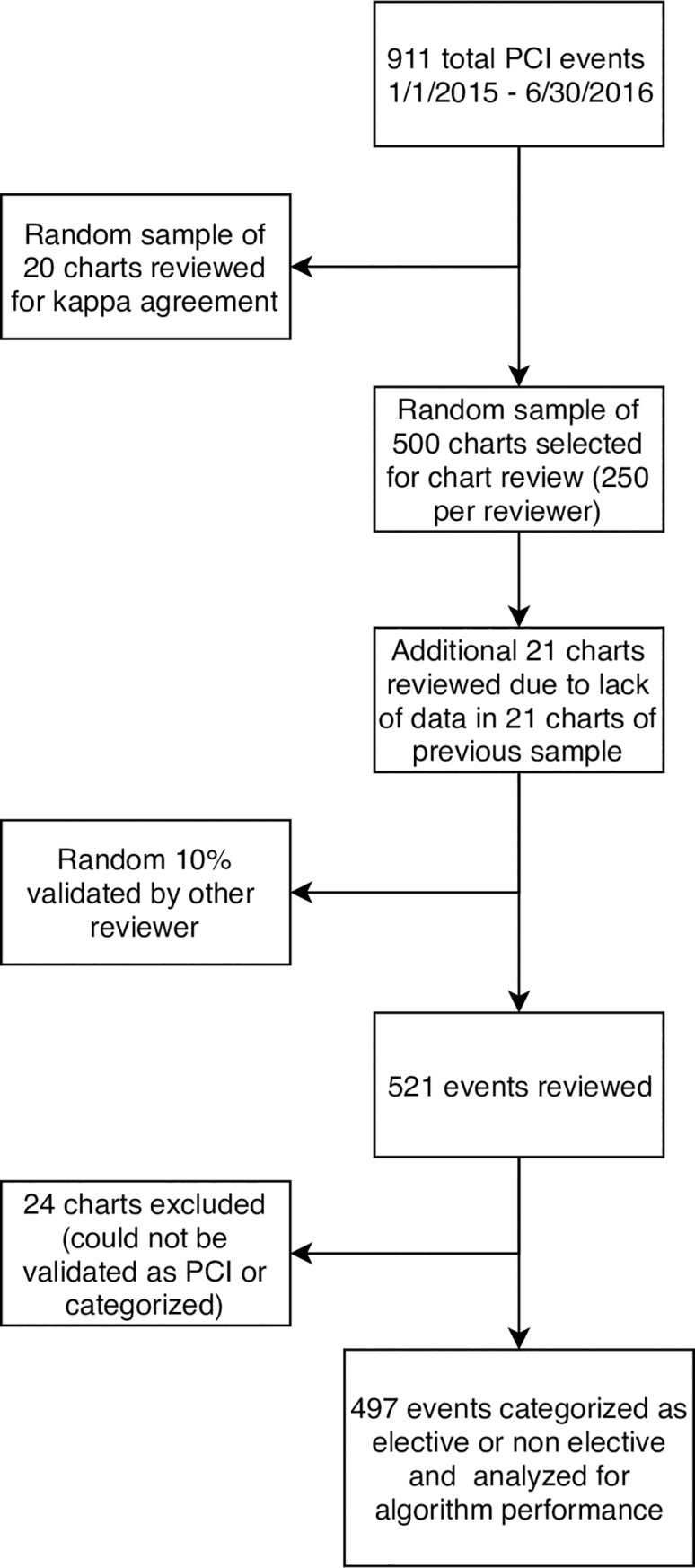
Development of cohort for analysis of algorithm performance. In the study period, 911 PCI events occurred. Of 521 events reviewed, 497 could be categorized as elective or non-elective and analyzed for algorithm performance. PCI = percutaneous coronary intervention.

There were 911 potential PCI identified during the study periods, and a random sample of 497 procedures were categorized as elective or non-elective (Fig 2). Ninety-three (18.7%) and 404 (81.3%) were determined to be elective and non-elective, respectively. Patients with an elective PCI were more likely to be male, have comorbid hypertension or diabetes, and have a higher mean Chronic Disease Score (Table 2, p < 0.05 for all). Patients with a non-elective PCI were more likely to have had a previous myocardial infarction (p = 0.007).

**Table 2:** Patient characteristics by elective and non-elective PCI status.

Characteristic	Elective PCI (n = 94)	Non-Elective PCI (n = 403)	p-value
Age, years	65.2 (± 10.3)	66.1 (± 12.2)	0.690
Female sex	19 (20.2)	124 (30.8)	0.042
Race			0.502
White	65 (69.2)	289 (71.7)	
Other	7 (7.5)	39 (9.7)	
Undeclared/Unknown	22 (23.4)	75 (18.6)	
Hispanic ethnicity			0.708
Hispanic	10 (10.6)	37 (9.2)	
Non-Hispanic	70 (74.5)	316 (78.4)	
Undeclared/unknown	14 (14.9)	50 (12.4)	
Tobacco use			0.161
Current	10 (10.6)	64 (15.9)	
Former	31 (33.0)	130 (32.3)	
Never	35 (37.2)	163 (40.5)	
Unknown/missing	18 (19.2)	46 (11.4)	
BMI, kg/m^2^	29.8 (± 4.2)	29.3 (± 5.9)	0.236
Median income, US Dollars ($)	66,752 (± 20,064)	62,808 (± 22,043)	0.584
Some college education	0.68 (± 0.15)	0.64 (± 0.18)	0.432
Chronic Disease Score	4.6 (± 3.9)	3.4 (± 3.4)	0.016
Charlson Comorbidity Index	2.2 (± 2.2)	2.1 (± 2.4)	0.398
Comorbidities			
Congestive heart failure	15 (16.0)	72 (17.9)	0.661
Peripheral vascular disease	22 (23.4)	67 (16.6)	0.123
Previous myocardial infarction	26 (27.7)	172 (42.7)	0.007
Hypertension	63 (67.0)	220 (54.6)	0.028
Diabetes	38 (40.4)	110 (27.3)	0.012
Previous PCI	7 (7.5)	32 (7.9)	0.873

All values are mean (±SD), median [IQR], or no. (%) unless noted otherwise.

BMI = body mass index; IQR = interquartile range; PCI = percutaneous coronary intervention; SD = standard deviation

The nine algorithms varied regarding performance measures (Table 3). Sensitivity ranged from 5.3% (algorithm 1) to 97.9% (algorithm 9), PPV ranged from 1.5% (algorithm 1) to 62.2% (algorithm 7), specificity ranged from 5.7% (algorithm 9) to 92.3% (algorithm 7), and NPV ranged from 57.8% (algorithm 1) to 97.0% (algorithm 3). Algorithm 9 had the highest sensitivity (97.9%, 95% CI 92.5%, 99.5%), although its PPV and specificity were low (19.5% and 5.7%, respectively). Algorithm 7 had the highest PPV (62.2%, 95% CI 50.8%, 72.7%). Algorithm 7 also had the highest specificity (92.3%, 95% CI 89.3%, 94.7%). Algorithm 3 had the highest NPV (97%, 95% CI 94.5%, 98.5%). Algorithm 3 also had high sensitivity (89.4%) and specificity (79.2%), although its PPV was low (50.0%). Algorithm 4 had similar performance statistics to Algorithm 3. Algorithms 1, 2, and 9 had the highest counts of false positives (Table 4). Algorithms 1, 2, and 7 had the highest counts of false negatives.

**Table 3:** Algorithm performance statistics*.

Algorithm No.	Sensitivity % (95% CI)	PPV % (95% CI)	Specificity % (95% CI)	NPV % (95% CI)
1	5.3 (1.8, 12.0)	1.5 (0.5, 3.4)	83.9 (12.7, 20.1)	57.8 (34.3, 50.4)
2	41.5 (31.4, 52.1)	12.6 (3.1, 16.9)	33.0 (28.4, 37.8)	70.7 (63.7, 77.1)
3	89.4 (81.3, 94.8)	50.0 (42.2, 57.8)	79.2 (74.9, 83.0)	**97.0 (94.5, 98.5)**
4	85.1 (76.3, 91.6)	49.1 (41.2, 57.0)	79.4 (75.1, 83.3)	95.8 (93.1, 97.7)
5	58.5 (47.9, 68.6)	57.3 (46.8, 67.3)	89.8 (86.5, 92.6)	90.3 (86.9, 93.0)
6	80.9 (74.4, 88.2)	22.7 (18.3, 27.6)	35.7 (31.1, 40.7)	88.9 (83.0, 93.3)
7	54.3 (43.7, 64.6)	**62.2 (50.8, 72.7)**	**92.3 (89.3, 94.7)**	89.6 (86.3, 92.0)
8	57.5 (46.8, 67.6)	47.8 (38.3, 57.4)	85.4 (81.5, 88.67)	89.6 (86.1, 92.5)
9	**97.9 (92.5, 99.7)**	19.5 (16.0, 23.4)	5.7 (3.7, 8.4)	92.0 (74.0, 99.0)

*Bolded values represent highest measure per column.

CI = confidence interval; NPV = negative predictive value; PPV = positive predictive value

**Table 4:** Counts of true positives, false positives, false negatives, and true negatives for each algorithm.

		Chart Review (Gold Standard) Performance	
		Elective N = 94	Non-Elective N = 403
**Algorithm Performance**	Elective	True positives	False positives
		Algorithm 1 = 5	Algorithm 1 = 338
		Algorithm 2 = 39	Algorithm 2 = 270
		Algorithm 3 = 84	Algorithm 3 = 84
		Algorithm 4 = 80	Algorithm 4 = 83
		Algorithm 5 = 55	Algorithm 5 = 41
		Algorithm 6 = 76	Algorithm 6 = 259
		Algorithm 7 = 51	**Algorithm 7 = 31**
		Algorithm 8 = 54	Algorithm 8 = 59
		**Algorithm 9 = 92**	Algorithm 9 = 380
	Non-Elective	False negatives	True negatives
		Algorithm 1 = 89	Algorithm 1 = 65
		Algorithm 2 = 55	Algorithm 2 = 133
		Algorithm 3 = 10	Algorithm 3 = 319
		Algorithm 4 = 14	Algorithm 4 = 320
		Algorithm 5 = 39	Algorithm 5 = 362
		Algorithm 6 = 18	Algorithm 6 = 144
		Algorithm 7 = 43	**Algorithm 7 = 372**
		Algorithm 8 = 40	Algorithm 8 = 344
		**Algorithm 9 = 2**	Algorithm 9 = 23

*Bolded values represent the most desirable measure per column (high values for true positives and true negatives, low values for false positives and false negatives).

Table 5 shows the results of the sensitivity analyses for algorithms that used diagnosis codes. The performance statistics were similar between ICD-9 and ICD-10 codes for algorithm 1. For algorithms 3 and 4, specificity was higher with ICD-10 codes compared to ICD-9 codes.

**Table 5:** Algorithm performance statistics by ICD-9/10.

Algorithm No.	Sensitivity Analysis	Sensitivity % (95% CI)	PPV % (95% CI)	Specificity % (95% CI)	NPV % (95% CI)
1	ICD-9 only	5.8	1.8	17.4	41.0
	(n = 247)	(1.2, 16.0)	(0.4, 5.3)	(12.4, 23.5)	(30.3, 52.3)
	ICD-10 only	4.8	1.1	14.9	43.7
	(n = 250)	(0.6, 16.2)	(0.1, 4.0)	(10.4, 20.5)	(31.9, 56.0)
3	ICD-9 only	**92.3**	48.5	73.9	**97.3**
	(n = 247)	**(81.5, 97.9)**	(38.3, 58.8)	(67.1, 79.9)	**(93.2, 99.3)**
	ICD-10 only	85.7	**52.2**	84.1	96.7
	(n = 250)	(71.5, 94.6)	**(39.8, 64.4)**	(78.5, 88.8)	(92.9, 98.8)
4	ICD-9 only	88.5	47.4	73.9	96.0
	(n = 247)	(76.6, 95.7)	(37.2, 57.8)	(67.1, 79.9)	(91.5, 98.5)
	ICD-10 only	81.0	51.5	**84.6**	95.7
	(n = 250)	(65.9, 91.4)	(38.9, 64.0)	**(79.0, 89.2)**	(91.6, 98.1)

*Bolded values represent highest measure per column.

CI = confidence interval; NPV = negative predictive value; PPV = positive predictive value

## Discussion

This retrospective analysis of patients from an integrated healthcare delivery system who underwent a PCI procedure identified several algorithms, which use diagnostic, utilization, and/or procedural codes that reasonably distinguish between elective and non-elective PCI. While the algorithms tested varied substantially in their ability to identify an elective PCI, an algorithm that excluded PCI as a part of an emergency department visit had the highest sensitivity (97.9%) to identify an elective PCI. An algorithm that assessed for an outpatient cardiology provider visit and either a stress test or angiography CPT code in the prior 90 days had the highest PPV (62.2%). To our knowledge, our study is the first to validate methods to identify patients who have had an elective PCI using administrative data. Our findings are important because providing a sensitive means to identify an elective PCI cohort represents a substantial step forward in methodology to measure the frequency of elective PCI, describe patient outcomes from elective PCI, and develop models predictive of patients requiring elective PCI. In addition, our findings may enable comparative analyses to assess specifically the outcomes of elective PCI in real-world settings.

Other investigations have used algorithms that combine encounter codes to identify a cardiovascular procedure. Davis and colleagues reported that a combination of ICD-9 and CPT codes resulted in a 53% sensitivity and 100% PPV to identify any PCI in veterans with rheumatoid arthritis even though PCI procedures were rare (prevalence = 2%) [24]. Another study designed to identify a revascularization procedure in patients receiving statin medications reported that a combination of CPT codes for coronary artery bypass grafting, angioplasty, and stenting had a PPV of 90–97% [25]. Interestingly, the majority of the CPT codes used in these studies are no longer available for use in the medical billing field (i.e., 92980–92982, 92984, and 92995–92996). To our knowledge, only one other study utilized place of service to identify an elective PCI population in a national claims database using discharge status and admission status, which can be classified as “outpatient’ or “inpatient,” and “elective” or “nonelective,” respectively [26]; the primary limitation with this unvalidated approach would be potential for misclassification bias. Further, none of our algorithms had a PPV greater than 62%, suggesting that current CPT codes were not developed in consideration of elective PCI. Development of CPT code(s) specific to elective PCI would be the most effective way to document these procedures and allow accurate identification of patients who received an elective PCI. Until such code(s) are available, our methodology provides a pragmatic approach to identifying elective PCI in administrative data.

The differences in performance statistics between algorithms observed in our study may reflect the complexity of the components included in each algorithm. CPT codes were the underlying mechanism for each algorithm, and algorithms 1–9 added levels of complexity through the addition of timing parameters, other healthcare utilizations (e.g., hospitalizations, inpatient admissions, diagnostic procedures), and diagnosis codes. Increasing the level of complexity narrows a target population, thereby reducing the potential to detect true positives, which may be particularly hard to detect for conditions with a low prevalence. Algorithm 9 was one of the simplest algorithms that we tested, perhaps indicating why it had the highest sensitivity; however, given its simplicity, algorithm 9 also had a high rate of false positives, reducing PPV and specificity. The selection of an algorithm should be based on the desire to increase true positives (i.e., optimize sensitivity or PPV) or prevent true negatives (i.e., optimize specificity or NPV). Future investigators may improve upon our algorithms by attempting to minimize false positives and false negatives; therefore, the algorithms with the highest counts of these parameters may be difficult to further refine (i.e., algorithms 1, 2, and 9). Algorithms 4, 5, and 7 had relatively high global performance statistics (i.e., across all performance statistics) compared to the others and may serve as meaningful candidates for future refinement.

Analyses of clinical registries have estimated that elective PCI constitute between 29% and 80% of PCI performed [10,27–32]. In our study, we identified that approximately 19% of PCI procedures were elective. Our lower rate may be because our study assessed PCI procedures that occurred within a defined time period, while clinical registry data may contain procedures across numerous years. In addition, we used a contemporary cohort of patients who received a PCI. The results of the COURAGE trial in 2007 that demonstrated equivalent rates of death and myocardial infarction in patients who underwent optimal medical treatment alone or optimal medical treatment plus PCI [6] and the development of appropriate use criteria in 2009 [5] likely reduced the frequency of elective PCI. Furthermore, the large range of reported elective PCI rates could be representative of significant practice variation in PCI application across geographic regions of the United States [33]. Further research is needed to describe trends in elective PCI use, broadly or geographically, across time and patient populations.

Our study of 497 validated PCI tested an expansive set of algorithms to discriminate elective PCI using administrative data. While we identified an algorithm with high sensitivity, our study had limitations. Our findings are dependent upon the accurate recording of CPT codes by medical billing specialists. If specialists are not billing appropriately (e.g., incorrect place of service), our algorithms will likely have lower sensitivity. As we conducted chart review to validate the type and place of service of and other exposures around the PCI in our study, we are confident that our algorithms are accurate. Since performance statistics are affected by prevalence, a larger sample of validated elective PCI may alter the sensitivity, specificity, PPV, and NPV values we report. Future studies should be undertaken with larger sample sizes to validate our findings. Finally, we did not assess the accuracy of the individual PCI CPT codes or algorithms within subgroups of patients due to our low rate of elective PCI. This may be of interest for future research.

This retrospective analysis using administrative data found that an algorithm which excluded PCI occurring in an emergency department setting was sensitive to elective PCI. Other algorithms had higher specificity, PPV, and NPV. Investigators who wish to use our methodology should chose an algorithm based on their desired outcome. Without a CPT code(s) specific to elective PCI, our algorithms offer a reasonable approach to identify elective PCI in administrative data.
